# MLL1 promotes myogenesis by epigenetically regulating *Myf5*

**DOI:** 10.1111/cpr.12744

**Published:** 2019-12-15

**Authors:** Shufang Cai, Qi Zhu, Cilin Guo, Renqiang Yuan, Xumeng Zhang, Yaping Nie, Luxi Chen, Ying Fang, Keren Chen, Junyan Zhang, Delin Mo, Yaosheng Chen

**Affiliations:** ^1^ State Key Laboratory of Biocontrol School of Life Sciences Sun Yat‐sen University Guangzhou Guangdong China

**Keywords:** H3K4me3, MLL1, Myf5, myogenesis, proliferation

## Abstract

**Objectives:**

Mixed lineage leukaemia protein‐1 (MLL1) mediates histone 3 lysine 4 (H3K4) trimethylation (me3) and plays vital roles during early embryonic development and hematopoiesis. In our previous study, we found its expression was positively correlated with embryonic myogenic ability in pigs, indicating its potential roles in mammalian muscle development. The present work aimed to explore the roles and regulation mechanisms of MLL1 in myogenesis.

**Materials and methods:**

The expression of MLL1 in C2C12 cells was experimentally manipulated using small interfering RNAs (siRNA). 5‐ethynyl‐2′‐deoxyuridine (EdU) assay, cell cycle assay, immunofluorescence, qRT‐PCR and Western blot were performed to assess myoblast proliferation and differentiation. Chromatin immunoprecipitation assay was conducted to detect H3K4me3 enrichment on myogenic factor 5 (*Myf5*) promoter. A cardiotoxin (CTX)‐mediated muscle regeneration model was used to investigate the effects of MLL1 on myogenesis in vivo.

**Results:**

MLL1 was highly expressed in proliferating C2C12 cells, and expression decreased after differentiation. Knocking down MLL1 suppressed myoblast proliferation and impaired myoblast differentiation. Furthermore, knockdown of MLL1 resulted in the arrest of cell cycle in G1 phase, with decreased expressions of Myf5 and Cyclin D1. Mechanically, MLL1 transcriptionally regulated *Myf5* by mediating H3K4me3 on its promoter. In vivo data implied that MLL1 was required for Pax7‐positive satellite cell proliferation and muscle repair.

**Conclusion:**

MLL1 facilitates proliferation of myoblasts and Pax7‐positive satellite cells by epigenetically regulating *Myf5* via mediating H3K4me3 on its promoter.

## 1 INTRODUCTION

Skeletal muscle is a heterogeneous and highly complex tissue that serves a multitude of necessary functions for animal survival.^1^ Myogenesis, including embryonic muscle development, postnatal growth and regeneration, is a highly coordinated event that depends on complex molecular regulatory networks. Myogenic regulatory factors (MRFs), including Myf5, myogenin, MyoD and Mrf4, are key transcription factors that play a central role in transcriptional regulation during muscle formation.^2^ During embryonic development, most myogenic cells are derived from dermomyotome. These cells are called progenitors and are marked by high expressions of the paired box transcription factors Pax3 and Pax7.^3^ Myf5 and MyoD are responsible for the specification of progenitors to committed myoblasts. Following this, myogenin and Mrf4 participate in the regulation of myoblast differentiation and fusion.^1^ In addition, the process of myoblast specification, proliferation, differentiation and fusion is controlled by many other transcription factors, including sine oculis–related homeobox 4 (Six4),^4^ double‐sex and mab‐3–related transcription factor 2 (Dmrt2),^5^ and nuclear factor I X (Nfix).^6^

In vertebrates, post‐translational modifications of histones, such as methylation, phosphorylation and acetylation, are extensively used to ensure the temporal and spatial expressions of key genes during tissue‐specific differentiation and development.^7^, ^8^ Lysine methylation on histone 3 (H3) is an important part of the epigenetic regulation network and integrates both cooperative and antagonistic modifications.^9^, ^10^ The trimethylation of histone 3 lysine 27 (H3K27me3) marks transcriptionally silenced genes,^11^ whereas trimethylation of histone 3 lysine 4 (H3K4me3) is generally associated with active gene expression.^12^ In addition, genome‐wide studies on chromatin states have revealed the existence of bivalent H3K4me3 and H3K27me3 chromatin domains that mark the silenced genes during development.^13^ Histone methylation is a dynamic change strictly regulated by histone methyltransferases and demethylases.^14^ Some modification enzymes of histone methylation, such as Utx histone demethylase (UTX),^15^ enhancer of zeste 2 (Ezh2) and zinc‐finger protein LSD1,^16^, ^17^ play crucial roles during myogenesis by mediating methylation or demethylation at muscle‐specific genes.

MLL1, a H3K4me3‐specific methyltransferase, is deemed as a transcriptional coactivator.^18^ It plays an important role in regulating gene expression during early embryonic development and hematopoiesis.^8^, ^19^ In humans, translocation and aberrant expression of MLL1 is observed in many tumours, thereby indicating its proto‐oncogenic character.^20^, ^21^, ^22^ A study in MLL1‐deficient mouse revealed that MLL1 is crucial for the function of hematopoietic progenitor cells.^7^ MLL1 is also required for the development of the central nervous system. Disrupting its expression resulted in neurogenic phenotypes, including decreased proliferation of neural progenitors and premature differentiation of neurons.^23^ Furthermore, MLL1 is indispensable for the development of visual system^24^ and greatly impacts neuronal signal processing and cognition.^25^ However, its role in myogenesis is unclear.

In our previous RNA sequencing results, MLL1 expression gradually increased during embryonic muscle development in three pig breeds differing in muscle mass, and its mRNA level was higher in the miniature pig breed, indicating its potential role in mammalian muscle development. Hence, the present study was designed to investigate the roles and regulatory mechanisms of MLL1 in myogenesis.

## 2 MATERIALS AND METHODS

### 2.1 Mice and harvest of embryos

Eight‐week‐old female and male mice (C57BL/6) were purchased from Guangdong Medical Laboratory Animal Center and were housed under specific‐pathogen‐free conditions. For timed pregnancies, mid‐day of vaginal plug detection was defined as embryonic day (E) 0.5. MLL1 expression profile during skeletal muscle development was determined using E9, E11, E13, E15, E17 and E18 embryos and mice 0.5 and 4 days post‐birth (P). For E9 and E11 embryos, dorsal muscle‐like tissues were isolated. For other embryos and neonatal mice, dorsal muscles were isolated. These tissues were cleaned in phosphate buffer saline (PBS) and were quickly frozen in liquid nitrogen for future experiments. All animal experiments were approved by the Animal Care and Use Committee of Guangdong Province and conducted according to ethical standards.

### 2.2 Cell culture

C2C12 cells, purchased from the American Type Culture Collection (ATCC), were cultured in Dulbecco's modified Eagle medium (DMEM) with 10% (v/v) foetal bovine serum (growth medium, GM). To induce differentiation, cells were switched into DMEM with 2% horse serum (differentiation medium, DM) after reaching 100% confluence. All cells were cultured in a 37°C incubator with 5% CO_2_.

### 2.3 RNA interference and overexpression

For RNA interference, negative control siRNAs (si‐NC) and three stealth mouse MLL1 siRNAs were purchased from Invitrogen (Thermo Fisher Scientific). The sequences of three MLL1‐targeting siRNAs are listed in Table S1, two of which are efficient (Figure S1). si‐MLL1, a mixture of si‐MLL1‐1 and si‐MLL1‐3, was used in all of the following analysis. For Myf5 expression vector, the coding sequences (CDSs) of mouse *Myf5* gene were inserted into pcDNA3.1 vector (Invitrogen). C2C12 cells were seeded into 6‐ or 12‐well plates at 12 hours before treatment and then transfected with siRNAs or expression plasmids using Lipofectamine 3000 (Invitrogen). Transfections were performed at least in triplicate for each experiment.

### 2.4 RNA extraction and real‐time quantitative PCR

For cultured C2C12 cells and induced myotubes, total RNA was extracted using TRIzol Reagent (Invitrogen). For dorsal muscles of embryos, neonatal mice and regenerating tibialis anterior (TA) muscles, total RNA was extracted using an RNeasy Mini Kit (Qiagen). Then, cDNA was synthesized from 1 μg total RNA using StarScript II First‐strand cDNA Synthesis Mix (Genestar). Real‐time quantitative PCR (qPCR) analyses were performed on LightCycler 480 II (Roche) using Hieff qPCR SYBR Green Master Mix (Yeasen). GAPDH was used as an internal control for normalization. Primers used for qPCR are listed in Table S2.

### 2.5 Western blot

Protein extracts of cultured C2C12 cells or TA muscles were obtained using lysis buffer (150 mmol/L NaCl, 50 mmol/L Tris, 1% Triton X‐100, 1% sodium deoxycholate, 0.1% SDS, pH 8.0) supplemented with protease inhibitor phenylmethanesulfonyl fluoride (PMSF, Thermo Scientific). Total protein was electrophoresed on 8% or 10% (w/v) SDS‐PAGE and transferred onto PVDF membrane (Bio‐Rad). After being blocked with 4% bovine serum albumin (BSA) for 1 hour, the membranes were incubated with primary antibodies at 4°C overnight, followed by incubation with proper secondary antibodies. Blots were visualized using an enhanced chemiluminescence (ECL) detection kit (FDbio). Antibodies are listed in Table S3.

### 2.6 Intramuscular transfection of siRNAs

Intramuscular transfection of siRNAs was performed using an Entranster‐in vivo kit (Engreen). Reagent A was prepared by mixing 12.5 μL siRNA (si‐NC or si‐MLL1, 1 μg/μL) with 12.5 μL sterile saline. Reagent B was prepared by mixing 6.25 μL Entranster‐in vivo with 18.75 μL sterile saline. Reagent A and reagent B were mixed completely, and the mixture was incubated at room temperature for 15 minutes. Afterwards, the hindlimbs of 8‐week‐old female mice were cleaned with 75% alcohol. Then, the mixture containing si‐MLL1 was injected into the left TA muscles, and the mixture containing si‐NC was injected into the right TA muscles as a negative control.

### 2.7 Cardiotoxin injury

Cardiotoxin (CTX) (Sigma) was dissolved in sterile saline to a final concentration of 10 mmol/L. Eight‐week‐old female mice were anaesthetized using a ketamine‐xylazine cocktail, and the hindlimbs were cleaned with 75% alcohol. Then, using hypodermic syringes (BD Biosciences), 50 µL of 10 mmol/L CTX was injected into the left and right TA muscles which have been transfected with si‐MLL1 and si‐NC one day before, respectively. Regenerating TA muscles were isolated 3 and 10 days after CTX injection.

### 2.8 Immunofluorescence

C2C12 cells cultured in 12‐well plates or laser confocal Petri dishes were fixed in 4% paraformaldehyde for 10 minutes, followed by permeabilization in 0.5% Triton X‐100 for 15‐20 minutes. The cells were blocked with 4% BSA in Tris‐buffered saline with Tween (TBST) for 1 hour. Then, the cells were incubated with primary antibodies overnight at 4°C. Afterwards, the cells were washed in PBS thrice and incubated with secondary antibodies for 1 hour at room temperature. Finally, the cells were washed thrice in PBS, and the nuclei were counterstained with 4′,6‐diamidino‐2‐phenylindole (DAPI; 1:1000 in PBS). Antibodies are listed in Table S3. Immunostaining images were obtained via fluorescent reverse microscopy (Nikon).

### 2.9 5‐Ethynyl‐2′‐deoxyuridine assay

5‐Ethynyl‐2′‐deoxyuridine (EdU) assay was performed with an EdU Kit (RiboBio). C2C12 cells transfected with si‐NC or si‐MLL1 were seeded onto 12‐well plates and cultured in GM for 48 hours, and then switched into fresh DMEM medium supplemented with EdU (50 mmol/L) and incubated for 2 hours, followed by fixation, permeabilization and EdU staining with Apollo 567 (RiboBio). The cell nuclei were stained with DAPI (1:1000 in PBS). The proportion of EdU‐positive cells was determined using fluorescent reverse microscopy (Nikon).

### 2.10 Propidium iodide staining and flow cytometry analysis

C2C12 cells transfected with si‐NC or si‐MLL1 were cultured in GM. Two days later, cells were harvested, washed with PBS thrice and then fixed in 70% ice alcohol at 4°C overnight. After being rinsed in PBS, fixed cells were incubated in PBS with 20 μg/mL RNase A for 30 minutes at room temperature. The cells were washed thrice again, and resuspended cells were stained with Propidium iodide (PI) solution (10 mg propidium iodide, 200 mg sodium citrate, 0.5 mL Triton X‐100 and 129.6 mL PBS in 200 mL, pH 7.2‐7.6) for 30 minutes in the dark at room temperature. Finally, cell cycle detection was conducted by a BD FACSCalibur system (BD Biosciences), and data analysis was performed using FlowJo 7.6 as per the manufacturer's instruction, created a polygon gate to eliminate the influence of cell adhesion, and then, the sample subset was analysed using the tool “cell cycle” to get the per cent of G0/G1, S and G2/M cells.

### 2.11 Chromatin immunoprecipitation

C2C12 cells transfected with si‐NC or si‐MLL1 were cross‐linked with 1% formaldehyde for 8 minutes at room temperature. The fixing solution was added with glycine and incubated for 5 minutes. Cells were lysed in lysis buffer (5 mmol/L PIPES, pH 8.1, 85 mmol/L KCI, 0.5% Nonidet P‐40) with protease inhibitor cocktail (MedChemExpress). Cell lysates were sonicated by Bioruptor (Covaris) for 8 min to generate chromatin fragments of 200‐300 bp DNA. The clarified nuclear extracts were incubated with tri‐methyl‐histone H3 (Lys4) antibody or *IgG* as a negative control overnight with rotation at 4°C and immunoprecipitated with CHROMATIN IMMUNOPRECIPITATION (ChIP)‐Grade protein G magnetic beads (Cell Signalling Technology). The enrichment of DNA sequences was analysed via quantitative PCR (qPCR) using specific *Myf5* primers, with *IgH* as a negative control. The primers are listed in Table S4. Data were normalized to the respective control *IgG* values and assessed relative to the input DNA.

### 2.12 Immunohistochemistry

Freshly isolated regenerating TA muscles were fixed in 4% paraformaldehyde at 4°C for 12 hours, dehydrated by graded ethanol and embedded in paraffin. Paraffin‐embedded samples were cut into 5‐μm sections using HM 340 rotary microtome (Microm) according to the manufacturer's instruction. Paraffin sections of TA muscle were dewaxed in xylene, rehydrated by graded ethanol and analysed by immunostaining with Pax7, Ki67 and embryonic myosin heavy chain (eMyHC) antibodies (listed in Table S3) using mouse‐on‐mouse polymer immunohistochemical kit (Abcam) as per the manufacturer's instruction. Images were captured by laser scanning confocal microscope (Leica). The myofiber diameters were quantified via Image‐Pro Plus6 software.

### 2.13 Statistical analysis

All experiments in this study were performed at least in triplicate. Data are presented as mean ± SEM, and the statistical significance analysis was performed using an unpaired two‐tailed Student's *t* test to test differences between groups. Values of *P* < .05 were considered as statistical significance.

## 3 RESULT

### 3.1 The expression pattern of MLL1 during muscle development

**Figure 1:**
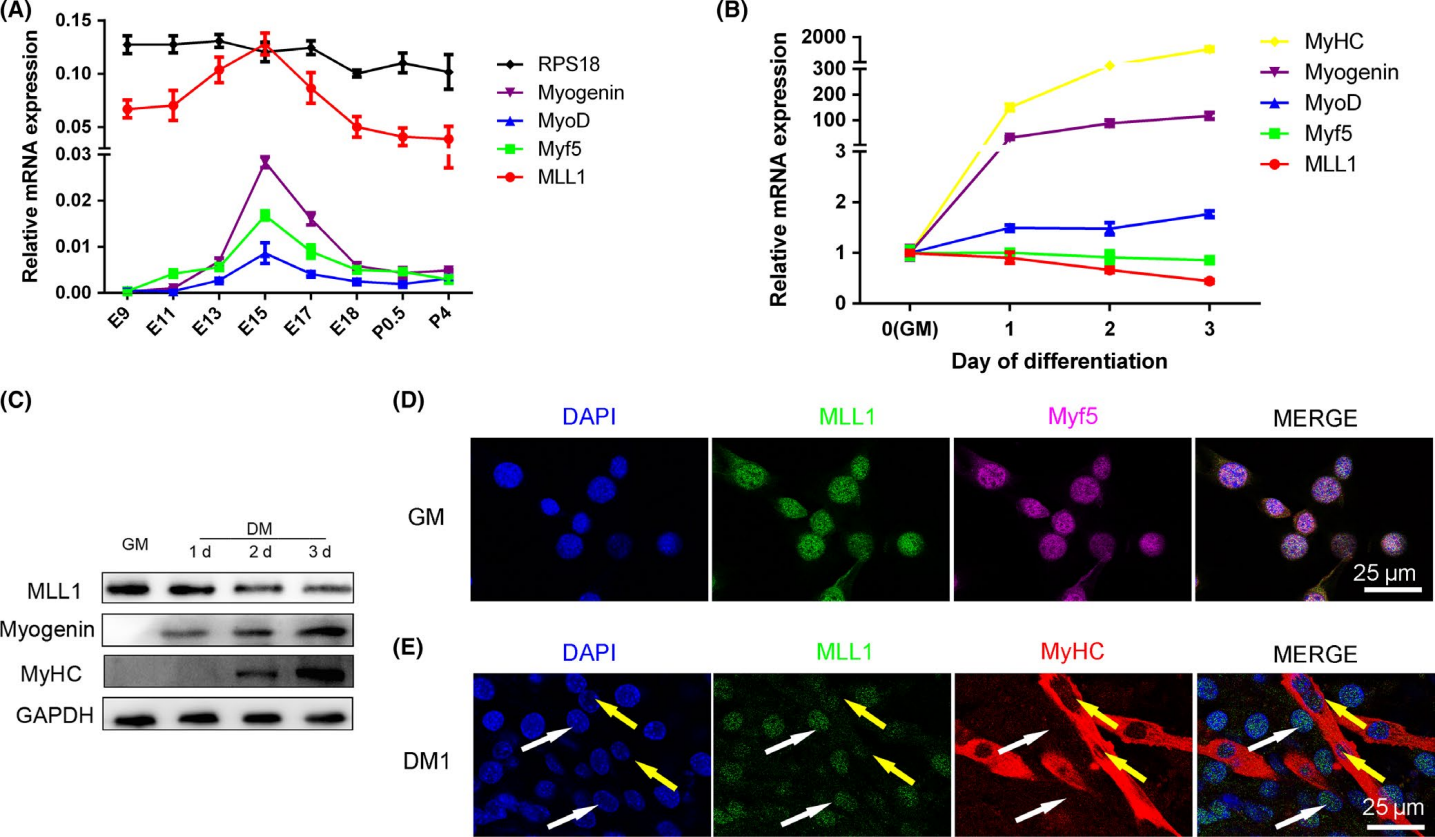
Expression pattern of MLL1 during muscle development. A, qPCR measurement of mRNA expression of MLL1 and myogenic markers (Myf5, MyoD and Myogenin) in dorsal muscle of mouse at several developmental times. GAPDH was used as an internal control for normalization, and ribosomal protein S18 (RPS18) was used as a negative control. E: days of embryo age, P: days of age post‐birth. B, The mRNA levels of MLL1 and myogenic markers during C2C12 cell differentiation at several indicated time points. When the cells were cultured in growth medium (GM) at sub‐confluent densities, it was defined as day 0; when the cells reached 100% confluence, GM was changed to differentiation medium (DM). C, Western blot analysis of MLL1 protein expression was performed in C2C12 cells grown in GM, as well as 1d, 2d and 3d in DM. Western blot for the myogenic markers myogenin and MyHC was performed to ensure that myogenic differentiation occurred properly. GAPDH was used as a loading control. D, Immunofluorescence staining of MLL1 and Myf5 (a marker of proliferating myoblasts) in C2C12 cells cultured in GM. The cell nucleus was stained with DAPI. Scale bar = 25 μm. E, Immunofluorescence staining of MLL1 and MyHC (a marker of differentiated myotubes) in C2C12 cells differentiated for 1 day in DM. White arrows indicate nucleus of myoblasts, and yellow arrows indicate nucleus in MyHC^+^ myotubes. Scale bar = 25 μm. Data are presented as mean ± SEM, n = 3 per group

We examined the mRNA level of MLL1 in the dorsal muscle of wild‐type mouse embryos at several developmental stages to determine the expression pattern of MLL1 during muscle development. As shown in Figure 1A, *MLL1* had the same expression trend as the key myogenic genes, such as *MyoD* and *Myf5*, indicating its potential role in embryonic myogenesis. We then explored the expression profile of MLL1 during myoblast differentiation. qPCR and Western blot results demonstrated that MLL1 was highly expressed during C2C12 cell proliferation but gradually decreased after differentiation. This expression was contrary to the expression of myogenin and myosin heavy chain (MyHC) (Figure 1B and C). This result was further confirmed by immunofluorescence staining of MLL1 and Myf5 (a marker of proliferating myoblasts) in C2C12 cells cultured in GM, in which MLL1 was highly expressed in Myf5‐positive myoblasts (Figure 1D). One day after differentiation, few MLL1 proteins were detected in MyHC‐positive myotubes, but many MLL1 proteins were detected in MyHC‐negative myoblasts (Figure 1E). These results suggested that MLL1 plays an important role in the proliferation stage of myoblasts.

### 3.2 MLL1 regulates myoblast proliferation through Cyclin D1

**Figure 2:**
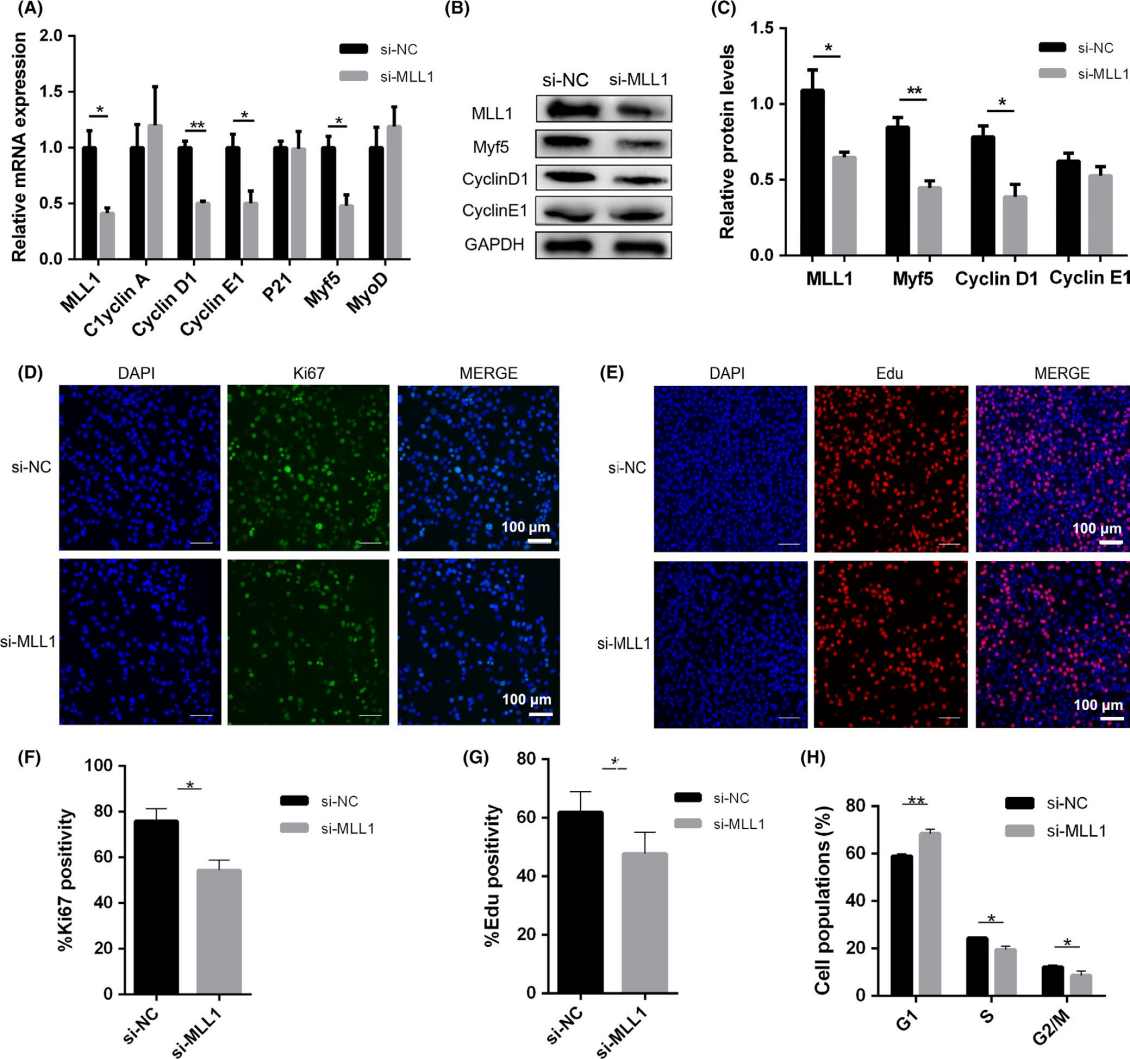
MLL1 regulates myoblast proliferation through Cyclin D1. C2C12 cells transfected with negative control siRNAs (si‐NC) or MLL1 siRNAs (si‐MLL1) were cultured in GM for 2 d. A, qPCR analysed the mRNA levels of MLL1, cell cycle regulators (Cyclin A/D1/E1, P21 and DHFR) and two myogenic transcription factors (MyoD and Myf5). B, Western blot detected the protein levels of MLL1, Myf5, Cyclin D1 and Cyclin E1 in proliferating C2C12 cells treated as above. C, The relative protein levels of target proteins normalized to GAPDH signals in (B) were obtained through Western blot (WB) band grey scanning. D, Immunofluorescent staining of Ki67 (a marker of proliferation) was performed to examine proliferation ability of si‐MLL1 C2C12 cells. Scale bar = 100 μm. E, Representative images of the EdU staining for si‐MLL1 C2C12 cells. Scale bar = 100 μm. F‐G, The percentages of EdU‐ and Ki67‐positive cells compared with the total number of nuclei were presented, respectively. H, The cell cycle phase of si‐NC and si‐MLL1 C2C12 cells was examined through flow cytometry analysis after PI staining. Data are presented as mean ± s.e.m., n = 3; *P < .05, **P < .01 (Student's *t* test)

C2C12 cells were cultured in GM and transfected with si‐MLL1 to knockdown endogenous MLL1 and determine the role of MLL1 during myoblast proliferation (Figure 2A and B). Two days later, EdU assay and Ki67 (a marker of cell proliferation) immunofluorescent staining were performed to detect the capacity of C2C12 proliferation (Figure 2D and E). As expected, the percentages of EdU‐ and Ki67‐positive cells decreased after knockdown of MLL1 (Figure 2F and G). Flow cytometry analysis after PI staining was conducted to clarify the mechanism of affecting proliferation. As shown in Figure 2H, knockdown of MLL1 prominently increased the population of cells in G1 phase, whereas S and G2/M populations remarkably declined. An arrest of cell cycle at G1 phase occurred in response to the decrease in MLL1 in C2C12 myoblasts.

The expression of myogenic factors (Myf5, MyoD), cyclins involved in G1/S transition and cyclin‐dependent kinase inhibitor 1A (P21) were detected via qPCR to address the molecular mechanism underlying the control of cell cycle progression through MLL1. The mRNA levels of Myf5, Cyclin D1 and Cyclin E1 were significantly reduced in si‐MLL1 groups (Figure 2A and S2A). Western blot proved that the protein levels of Myf5 and Cyclin D1 also declined, whereas Cyclin E1 protein level was not be changed (Figure 2B, 2C and S2B). Based on these results, we conclude that MLL1 maintains myoblast proliferation by controlling the expression of Myf5 and Cyclin D1.

### 3.3 MLL1 regulates the expression of Myf5 by H3K4me3 modification

Myf5 is the first MRF expressed during myogenesis and functions as a transcription factor in muscle progenitor cells and myoblasts.^26^ In mouse C2C12 cells, silencing *Myf5* impaired myoblast proliferation and differentiation. Myf5 enhances early myogenesis by elevating Cyclin D1 expression.^27^ In the present study, MLL1 knockdown inhibited myoblast proliferation and reduced the expressions of Myf5 and Cyclin D1. These results promoted us to hypothesize that MLL1 regulates myoblast proliferation by directly regulating the expression of Myf5, thereby further affecting Cyclin D1 expression.

**Figure 3:**
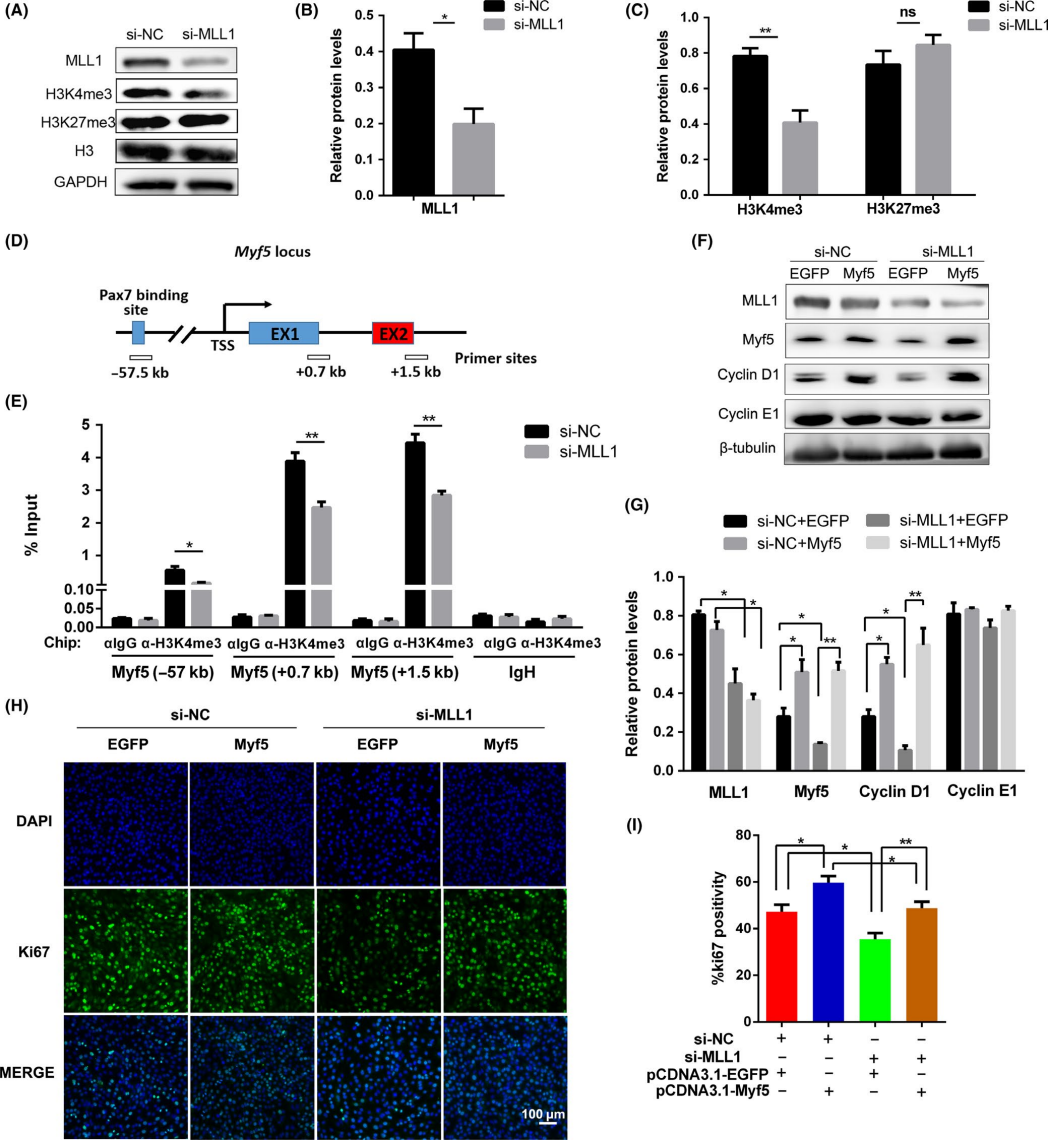
MLL1 regulates Myf5 expression through H3K4me3 modification. A, Western blot detected the levels of MLL1, H3K4me3 and H3K27me3 in si‐MLL1 cells cultured in GM. GAPDH and histone H3 (H3) were used as loading control. B, The relative protein levels of MLL1 normalized to GAPDH signals in (A) were obtained through WB band grey scanning. C, The relative levels of histone H3K4me3 and H3K27me3 normalized to H3 signals in (A). D, Location of primer sets for ChIP‐qPCR analysis of *Myf5* promoter. TSS: transcription start site. E, ChIP‐qPCR analysis of H3K4me3 enrichment on *Myf5* promoter in si‐MLL1 cells. *IgH* enhancer region was used as a negative control. Data were normalized as a percentage of the input. F, C2C12 cells, cotransfected with si‐NC or si‐MLL1 and pcDNA3.1‐EGFP or pcDNA3.1‐Myf5 vector, were cultured in GM for 48 h. The protein levels of MLL1, Myf5 and Cyclin D1 in four groups were detected by Western blot. β‐tubulin was used as a loading control. G, The relative protein levels of target proteins normalized to β‐tubulin signals in (F). H, C2C12 cells were treated as indicated in (F), and Ki67 immunofluorescent staining was performed to compare cell proliferation ability between four experiment groups. Scale bar = 100 μm. I, The percentage of Ki67‐positive cells in (H) were counted in six microscopic fields for each group. Data are showed as mean ± SEM, n = 6 per group. *P < .05, **P < .01 (Student's *t* test)

As a post‐translational modification factor, MLL1 usually contributes to the temporal and spatial expressions of key genes during development by mediating H3K4me3 modification. As predicted, the level of H3K4me3 was reduced in MLL1 knockdown groups, and the level of H3K27me3 was not changed (Figure 3A, B and C). To verify the possibility that MLL1 regulates the expression of Myf5 by mediating H3K4me3, ChIP analysis was performed. An antibody against H3K4me3 was used to pull down H3K4me3‐DNA complexes from si‐NC and si‐MLL1 cells. As a result, H3K4me3 was mostly enriched in the +0.7 kb and +1.5 kb regions (relative to the transcription start site) of *Myf5*, and the enrichment was reduced when MLL1 was knocked down (Figure 3D and E). Hence, these results suggest that MLL1 promotes *Myf5* transcription by increasing the enrichment of H3K4me3.

### 3.4 Myf5 rescued the effect of decreased MLL1 on myoblast proliferation

To further test whether the effect of MLL1 on myoblast proliferation was mediated by Myf5, pcDNA3.1‐EGFP or pcDNA3.1‐Myf5 vectors were transfected into C2C12 cells simultaneously treated with si‐NC or si‐MLL1. As predicted, Myf5 overexpression rescued the repressed expression of Cyclin D1 (Figure 3F and G) and the inhibited proliferation caused by MLL1 knockdown in C2C12 cells (Figure 3H and I). These results further proved that MLL1 maintains the proliferation capacity of C2C12 cells by regulating the expression of Myf5.

### 3.5 Knockdown of MLL1 impairs myogenic differentiation

**Figure 4:**
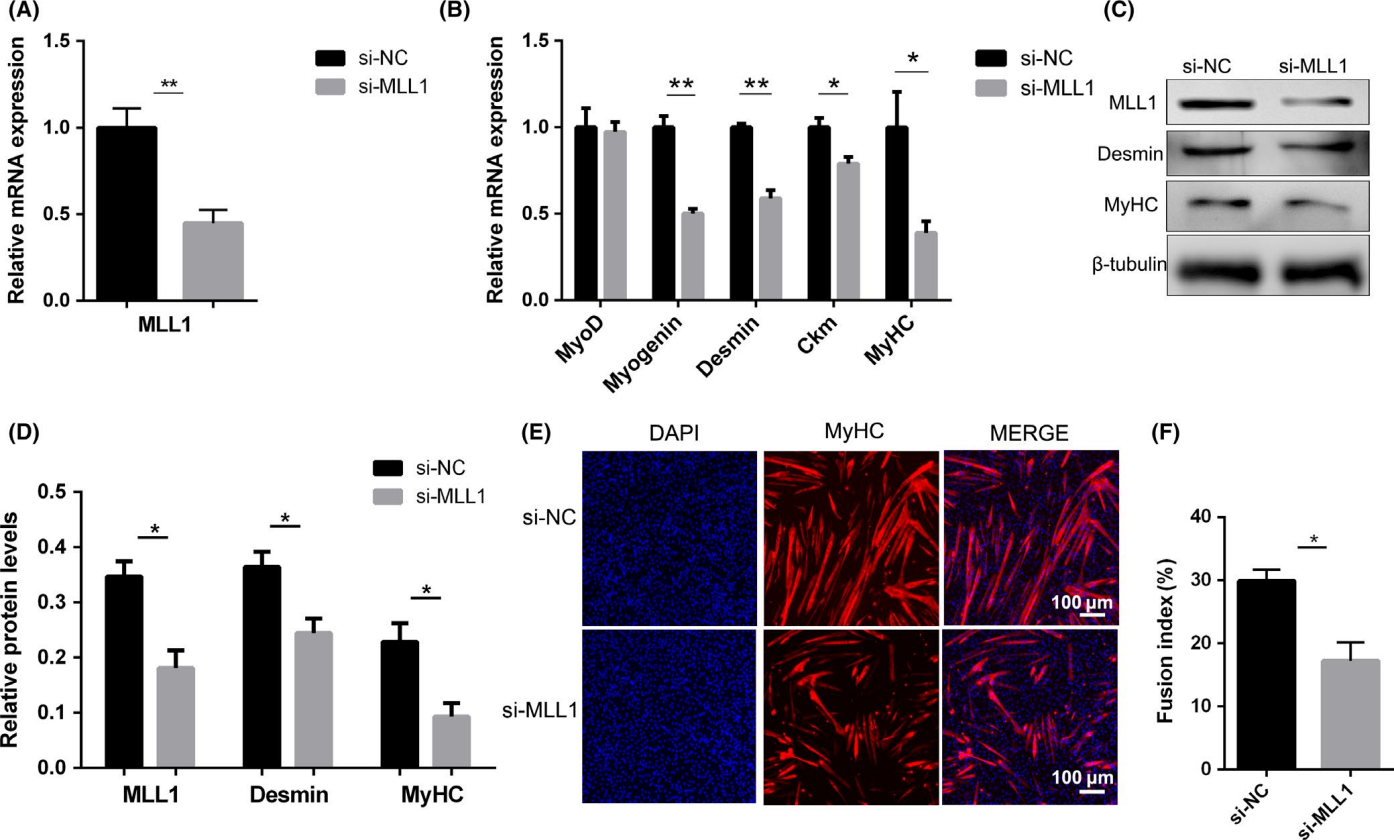
Knockdown of MLL1 impairs myogenic differentiation. C2C12 cells were transfected with si‐NC or si‐MLL1, and then induced to differentiate in DM for 3 days. A‐B, qPCR was performed to detect the mRNA levels of MLL1 as well as myogenic differentiation markers. C, Western blot detected the protein levels of MLL1, Myogenin and MyHC. β‐tubulin was used as a loading control. D, The relative protein levels of target proteins normalized to β‐tubulin signals in (C) were obtained through WB band grey scanning. E, Immunofluorescent staining for MyHC in si‐NC or si‐MLL1 C2C12 cells was performed to detect myotube formation. The cell nucleus was stained with DAPI. Scale bar = 100 μm. F, The fusion index (the percentage of nuclei in fused myotubes out of the total nuclei) in (E) was calculated. For each group, six random microscopic fields were selected randomly. Data are showed as mean ± SEM, n = 6 per group. *P < .05, **P < .01 (Student's *t* test)

C2C12 cells transfected with si‐NC or si‐MLL1 were induced to differentiate to evaluate whether MLL1 affected the differentiation of myoblasts (Figure 4A and C). The results showed that knockdown of MLL1 substantially reduced the mRNA levels of Myogenin, Desmin, creatine kinase (Ckm) and *MyHC* at 3 days after differentiation induction (Figure 4B). In addition, the protein levels of Desmin and MyHC also decreased considerably (Figure 4C and D). Consistently, MyHC immunofluorescence showed that myogenic differentiation was impaired, with lesser and smaller myotubes in response to the decrease in MLL1 (Figure 4E). Cell fusion index also declined markedly (Figure 4F), although the mRNA expression of cell fusion genes remained unchanged (Figure S3). In summary, knockdown of MLL1 impairs myogenic differentiation.

### 3.6 MLL1 knockdown impairs muscle regeneration

**Figure 5:**
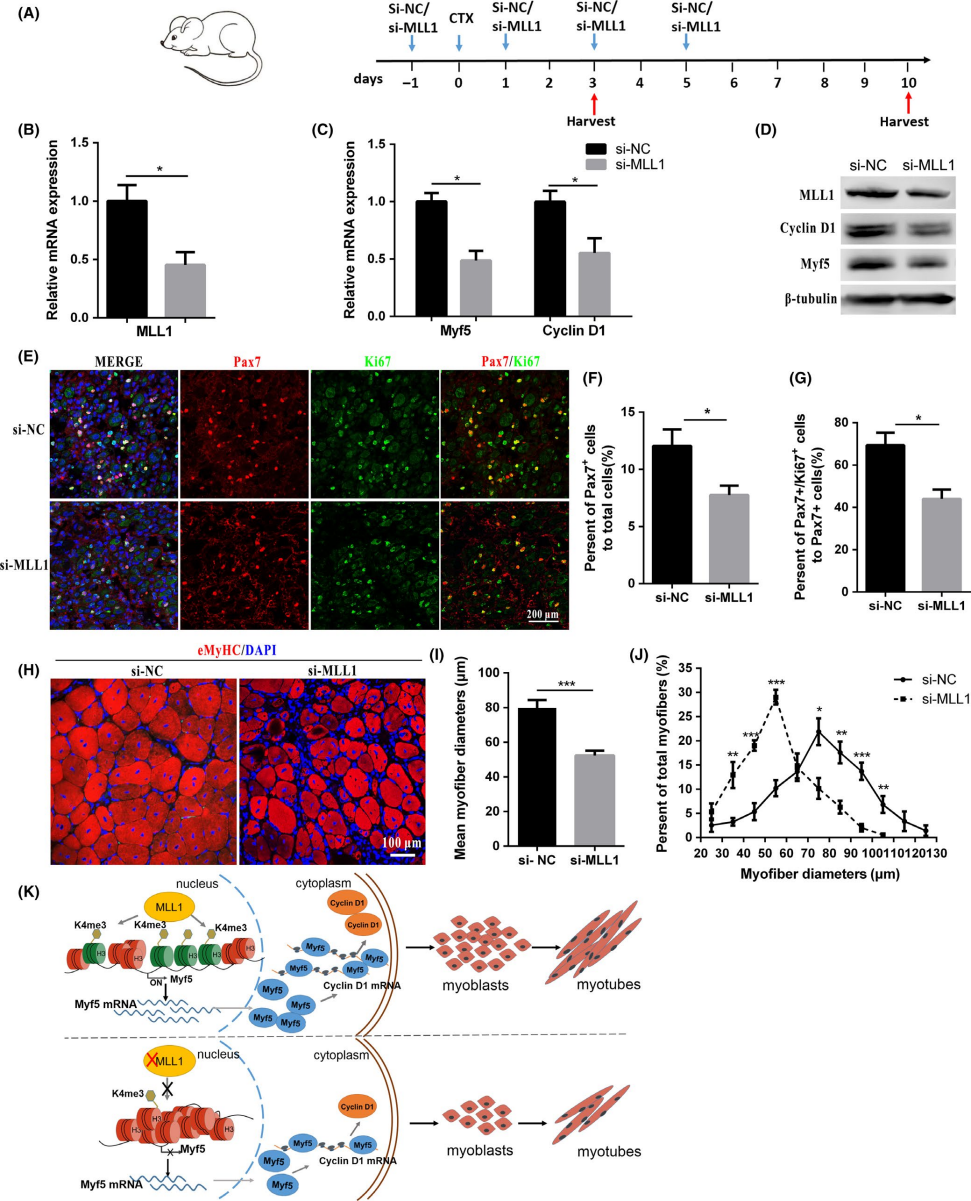
MLL1 deficiency impairs muscle regeneration. A, Schematic diagram of si‐MLL1–mediated MLL1 knockdown in the cardiotoxin (CTX) injury muscle model. When the tibialis anterior muscles (TA) were treated with CTX, it was defined as day 0. The left and right TA muscles were treated with si‐MLL1 and si‐NC, respectively. B‐C, The relative mRNA levels of MLL1, Myf5 and Cyclin D1 in regenerating TA muscles at day 3. D, The protein levels of MLL1, Myf5 and Cyclin D1 in regenerating TA muscles at day 3. E, Immunofluorescence staining of Pax7 (a marker of muscle satellite cells) and Ki67 on cross sections of regenerating TA muscles at day 3. Scale bar = 200 μm. F, Pax7‐positive cells in regenerating TA muscle sections were counted in six microscopic fields for each group at day 3. The percentage of Pax7‐positive cells compared with the total number of nuclei was presented. G, The percentage of Pax7/Ki67 double‐positive cells compared with Pax7‐positive cells in each group was presented at day 3. H, Immunofluorescence staining of eMyHC was performed on cross sections of regenerating TA muscles at day 10. Scale bar = 100 μm. I, Average myofiber diameters of regenerating TA muscles were measured at 10 d. More than 500 myofibers per group were analysed. J, Per cent distributions by diameter of myofibers were calculated. Data are presented as mean ± SEM, n = 6 per group. *P < .05, **P < .01, ***P < .001 (Student's *t* test). K, Schematic diagram of the mechanism by which the MLL1‐Myf5 axis modulates myogenesis. MLL1 activates Myf5 transcription through mediating H3K4me3 enrichment at the *Myf5* promoter. Then, Myf5 promotes Cyclin D1 expression. In MLL1 knockdown cells, Myf5 expression was down‐regulated due to reduced H3K4me3 enrichment on its promoter

A CTX‐mediated muscle regeneration model was used to determine whether the functions of MLL1 in C2C12 cells can be recapitulated in vivo. si‐MLL1 or si‐NC was injected into TA muscles every 2 days to maintain the efficiency of MLL1 knockdown. Then, regenerating TA muscles were harvested at days 3 and 10 (Figure 5A). mRNA and protein levels of Myf5 and Cyclin D1 were markedly down‐regulated in TA muscles administrated with si‐MLL1 at day 3 compared with controls (Figure 5B, C and D). This result is consistent with the in vitro experiments (Figure 2A and B). Immunofluorescence staining for Pax7 (a marker of satellite cells) and Ki67 on TA sections revealed that knockdown of MLL1 resulted in less Pax7‐positive cells (Figure 5E and F) and a decreased ratio of Pax7/Ki67 double‐positive cells to Pax7‐positive cells at day 3 (Figure 5G). Immunofluorescence of eMyHC on TA sections showed that MLL1 knockdown resulted in smaller myofibers at day 10 (Figure 5H). The mean myofiber diameters were remarkably reduced (Figure 5I), and the distributions of myofiber diameters shifted towards smaller diameters in si‐MLL1–treated TA sections (Figure 5J). Thus, intramuscular injection of si‐MLL1 inhibited the expansion of Pax7‐positive satellite cells through the down‐regulation of Myf5 and Cyclin D1, and subsequently blunted muscle regeneration. These results were consistent with the in vitro observations that knocking down MLL1 inhibited C2C12 proliferation, thereby leading to compromised myogenesis.

## 4 DISCUSSION

Myogenesis is a complex and well‐orchestrated biological process regulated through changes in gene expression programmes. MLL1, a major mammalian histone methyltransferase, is involved in the spatial and temporal expressions of genes during development by mediating H3K4me3. MLL1 is important for stemness, cell cycle progression and cell survival in hematopoiesis and nervous system development. In this study, we identified the involvement of MLL1 in myogenesis. Knockdown of MLL1 in C2C12 myoblasts inhibited myoblast proliferation and myotube formation.

Myf5 functions as a crucial transcription factor in muscle progenitor cells and myoblasts.^28^ In mouse C2C12 cells, Myf5 is implicated in cell proliferation and in the initial steps of myoblast differentiation.^29^, ^30^ Cyclin D1 is required for cell cycle G1/S transition. In addition, silencing Myf5 in proliferating myoblasts revealed that Myf5 promotes Cyclin D1 expression. 27 Thus, in our study, the si‐MLL1–mediated G1/S‐phase arrest might be due to the simultaneous reduction in Myf5 and Cyclin D1 proteins. Furthermore, we hypothesized that MLL1 probably regulates myoblast proliferation by directly regulating Myf5 expression, which further affects the expression level of Cyclin D1.

Histone methylation modifications are essential for the structure and function of chromatin and can regulate gene expression.^31^ The consequences of histone methylation on transcriptional repression or activation depend on the site and degree of methylation, which are mediated by a variety of methyltransferases and demethylases.^32^, ^33^ The expression of genes involved in muscle development is under the strict control of epigenetic mechanisms through histone methylation. For example, MLL3/MLL4‐mediated H3K4me1 on enhancers is important for the induction of cell identity gene in myogenesis.^9^ MyoD regulates the transcriptional initiation of downstream genes by recruiting epigenetic regulatory factors and forming positive histone methylation within promoter and enhancer regions.^34^ During myoblast proliferation, H3K4me3 marks on *Pax7* promoter contribute to the stability of Pax7 expression.^35^ Upon the initiation of myogenic differentiation, Per‐Arnt‐Sim (PAS) domain‐containing protein kinase stimulates the conversion of repressive H3K4me1 into activating H3K4me3 on the promoter of *myogenin*.^36^ *Ckm* gene is marked by Trithorax‐mediated H3K4me3 during myogenic differentiation, permitting its transcription.^37^

Whether methyltransferase MLL1 regulates *Myf5* transcription through an epigenetic mechanism was determined. The level of H3K4me3 in proliferating myoblasts was significantly down‐regulated when MLL1 was knocked down. However, H3K27me3 did not show any remarkable change. Methylations of H3K4 and H3K27 were mainly enriched in the transcriptional regulatory regions of genes. Results of ChIP‐qPCR assays revealed that MLL1 regulates *Myf5* transcription by mediating H3K4me3 on its promoter. In addition, Myf5 can rescue the effects of decreased MLL1 on myoblast proliferation. The up‐regulation of Cyclin D1 protein, which resulted from Myf5 overexpression, confirmed that Myf5 controlled myoblast proliferation by elevating Cyclin D1 expression.

Cell cycle withdrawal is required for the initiation of myogenic differentiation.^38^ We proved that MLL1 knockdown promotes cell exit from the cell cycle, by mediating G1/S‐phase arrest in GM. However, knocking down MLL1 inhibited the differentiation and fusion of myoblasts in DM, although the expression of cell fusion genes remained unchanged. Cell division of myoblasts is crucial for establishing cell‐to‐cell contacts initiating their differentiation. Silencing Myf5 inhibited C2C12 proliferation and their subsequent differentiation into myotubes.^32^ Therefore, the inhibited differentiation of si‐MLL1 myoblasts might also be mediated by the down‐regulation of Myf5 expression.

As a renewable organ, mature skeletal muscle has a high regeneration capacity which benefits from the presence of satellite cells involved in a series of cascade events, including quiescent satellite cell activation, satellite cell proliferation, myoblast differentiation and myocyte fusion.^39^ In this study, intramuscular injection of si‐MLL1 inhibited the expansion of Pax7‐positive satellite cells during CTX‐mediated muscle regeneration. Myf5 induction demarcates the entry of satellite cells into myogenic programme.^40^, ^41^ The lower number and weaker proliferation capacity of Pax7‐positive satellite cells were due to the decreased expression of Myf5 and Cyclin D1. Therefore, MLL1 is required for the activation of satellite stem cells and facilitates their expansion during muscle regeneration.

In summary, as illustrated in Figure 5K, our results highlight the important role of MLL1‐Myf5 axis in myogenesis. MLL1 is required for myoblast proliferation and differentiation. Further study revealed that MLL1 epigenetically regulates *Myf5* transcription by mediating H3K4me3, and indirectly affects the expression of Cyclin D1. Given the crucial role of Myf5 in muscle development and regeneration, our study contributes to the accumulation of theoretical basis for the prevention and treatment of muscle diseases.

## CONFLICT OF INTEREST

The authors have no competing interests.

## AUTHOR CONTRIBUTIONS

D.M. and Y.C. conceived and designed the experiments. S.C. designed and carried out most of the experiments, and manuscript writing. Q.Z. conducted Chip‐qPCR analysis. R.Y. provided analysis of sequencing data. C.G., K.C., Y.F. and J.Z. helped in cell culture and sample collecting. X.Z., L.C. and Y.N. advised on experimental design and participated in the analysis of data. All authors reviewed and approved this manuscript.

## Data Availability

All data, models and code generated or used during the study appear in the submitted article.
