# Supplementary material for: MLL1 promotes myogenesis by epigenetically regulating Myf5
Source: Cell Prolif. 2019 Dec 15;53(2):e12744. doi: 10.1111/cpr.12744 (PMC7046306; doi:10.1111/cpr.12744)
Supplement: Supplementary file 1 [file CPR-53-e12744-s001.docx]

**Fig S1. knockdown efficiency determination of si-RNAs against MLL1** 3 si-RNAs (si-MLL1-1, si-MLL1-2, si-MLL1-3) against MLL1 and control si-NC were transfected into C2C12 cells for 2 days respectively, and knockdown efficiency were determined by qPCR. Data are presented as mean ± S.E.M.; n=3; *p < 0.05, **p < 0.01, ***p < 0.001 (Student’s t test).


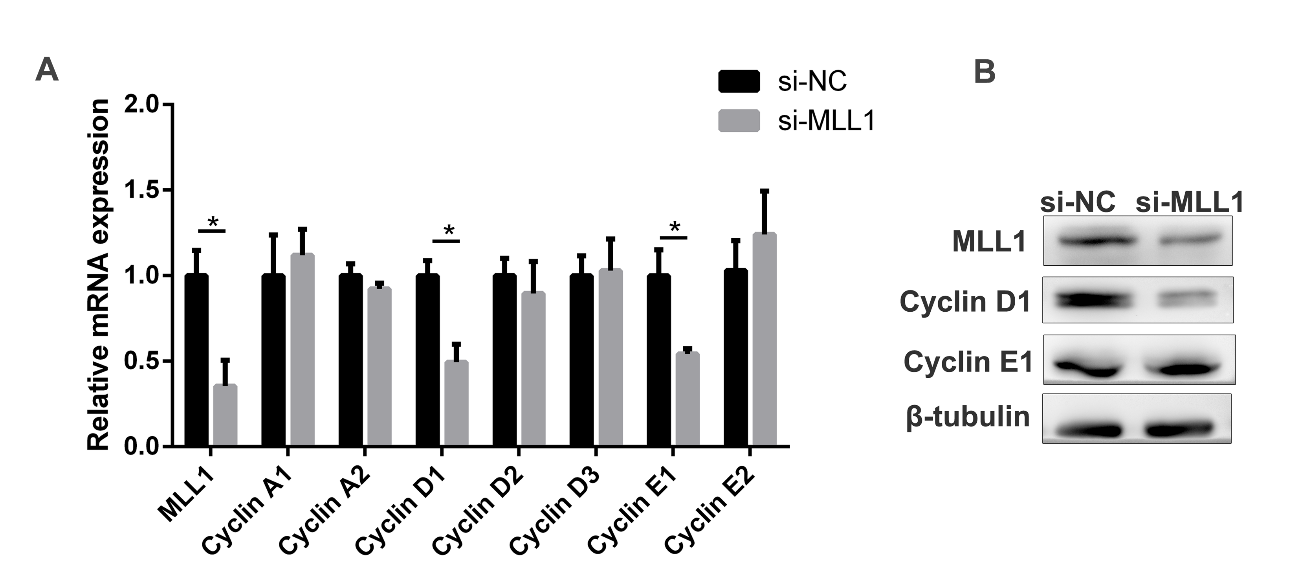


**Fig S2. MLL1 knockdown reduces the protein level of Cyclin D1 but not Cyclin E1.** C2C12 cells, which had been transfected with negative control siRNAs (si-NC) or MLL1 siRNAs (si-MLL1), were cultured in growth medium for 2 days. **(A)** qPCR analysed the mRNA levels of MLL1 and cyclins. GAPDH was used as an internal control. Data are presented as mean ± s.e.m., n= 3 per group. *p< 0.05 (Student’s t test). **(B)** Western blotting (WB) detected the protein levels of MLL1, Myf5, Cyclin D1 and Cyclin E1 in proliferating C2C12 cells treated as above.





**Fig S3. qPCR measurement of mRNA expression of genes involved in cell fusion.** qPCR was performed to detect the relative mRNA expression of genes involved in cell fusion in C2C12 cells transfected with si-NC or si-MLL1 and cultured in differentiation medium for 2 days. GAPDH was used as an internal control. Data are presented as mean ± s.e.m., n= 3 per group. ***p< 0.001 (Student’s t test).

| Table S1 The sequences of MLL1-targeting si-RNAs | |
| --- | --- |
| siRNA | Sequence |
| si-MLL1 -1 | F: GGU CAG GAU UAU UCC UUC UUG UAA A |
|  | R: UUU ACA AGA AGG AAU AAU CCU GAC C |
| si-MLL1 -2 | F: CCC AAG UUU GGU GGC CGC AAU AUA A |
|  | R: UUA UAU UGC GGC CAC CAA ACU UGG G |
| si-MLL1 -3 | F: UCA CAG ACC UCA GGC UCC UGU UAU U |
|  | R: AAU AAC AGG AGC CUG AGG UCU GUG A |

| Table S2 The primers for qPCR | | |
| --- | --- | --- |
| Gene | Sequences | |
| Myf5 | F: CCTGTCTGGTCCCGAAAGAAC | R: GACGTGATCCGATCCACAATG |
| MyoD | F: GCCTGAGCAAAGTGAATGAG | R: GCAGACCTTCGATGTAGCG |
| MyHC | F: AGAAGGAGGAGGCAACTTCTG | R: ACATACTCATTGCCGACCTTG |
| Myogenin | F: GCAATGCACTGGAGTTCG | R: ACGATGGACGTAAGGGAGTG |
| MLL1 | F: GCAGATTGTAAGACGGCGAG | R: GAGAGGGGGTGTTCCTTCCTT |
| GAPDH | F: CATGGCCTTCCGTGTTCCTA | R: TGCCTGCTTCACCACCTTCT |
| Ckm | F: CTGACCCCTGACCTCTACAAT | R: CATGGCGGTCCTGGATGAT |
| Myf5 | F: CCTGTCTGGTCCCGAAAGAAC | R: GACGTGATCCGATCCACAATG |
| Cyclin E1 | F: ATGTCAAGACGCAGCCGTTTA | R: GCTGATTCCTCCAGACAGTACA |
| Cyclin D1 | F: GCGTACCCTGACACCAATCTC | R: CTCCTCTTCGCACTTCTGCTC |
| Cyclin A | F: AAGAGAATGTCAACCCCGAAAAA | R: ACCCGTCGAGTCTTGAGCTT |
| P21 | F: CCTGGTGATGTCCGACCTG | R: CCATGAGCGCATCGCAATC |
| DHFR | F: CGCTCAGGAACGAGTTCAAGT | R: TGCCAATTCCGGTTGTTCAATA |
| Cyclin A1 | F: TGATGCTTGTCAAATGCTCAGC | R: AGGTCCTCCTGTACTGCTCAT |
| Cyclin A2 | F: GCCTTCACCATTCATGTGGAT | R: TTGCTGCGGGTAAAGAGACAG |
| Cyclin D2 | F: GAGTGGGAACTGGTAGTGTTG | R: CGCACAGAGCGATGAAGGT |
| Cyclin D3 | F: CGAGCCTCCTACTTCCAGTG | R: GGACAGGTAGCGATCCAGGT |
| Cyclin E1 | F: ATGTCAAGACGCAGCCGTTTA | R: GCTGATTCCTCCAGACAGTACA |
| RPS18 | F: AGTTCCAGCACATTTTGCGAG | R: TCATCCTCCGTGAGTTCTCCA |

| Table S3 Antibodies and their application | | | | |
| --- | --- | --- | --- | --- |
| Antibody name | Catalogue Number | Brand name | Dilution ratio | |
|  |  |  | WB | IF |
| Anti-rabbit IgG, HRP-linked Antibody | #7074 | Cell Signaling | 1/1000 |  |
| Anti-mouse IgG, HRP-linked Antibody | #7076 | Cell Signaling | 1/1000 |  |
| Anti-mouse IgG (H+L),F(ab')2 Fragment (Alexa Fluor 555 Conjugate) | #4409 | Cell Signaling |  | 1/1000 |
| Anti-rabbit IgG(H+L),F(ab')2 Fragment (Alexa Fluor 488 Conjugate) | #4412 | Cell Signaling |  | 1/1000 |
| Anti-Fast Myosin Skeletal Heavy chain Antibody | Ab91506 | abcam | 1/1000 | 1/500 |
| Anti-Ki67 antibody | ab15580 | abcam | 1/1000 | 1/500 |
| Anti-Myf5 antibody | ab125301 | abcam | 1/1000 |  |
| Anti-Myf5 antibody | 39801 | active motif |  | 1/100 |
| Anti-Myogenin Antibody | MAB3876 | Merck Millipore | 1/200 |  |
| Anti-Desmin antibody | sc-14026 | Santa Cruz Biotechnology | 1/200 |  |
| Anti-GAPDH antibody | ab8245 | abcam | 1/1000 |  |
| Anti-Histon H3.3 antibody | ab176840 | abcam | 1/1000 |  |
| Anti-MLL1 antibody | 14197s | Cell Signaling | 1/500 | 1/100 |
| Tri-Methyl-Histone H3 (Lys4) (C42D8) Rabbit mAb | 9751T | Cell Signaling | 1/1000 |  |
| Tri-Methyl-Histone H3 (Lys27) (C36B11) Rabbit mAb | #9733 | Cell Signaling | 1/1000 |  |
| Anti-Cyclin D1 antibody | 2978T | Cell Signaling | 1/1000 |  |
| β-Tubulin Antibody | #2148 | Cell Signaling | 1/1000 |  |
| Anti-MyoD antibody | ab212662 | abcam | 1/1000 | 1/200 |
| Anti-Pax7 antibody | PA1117 | thermo | 1/1000 | 1/200 |

| Table S4 PCR primers for ChIP-qPCR | | |
| --- | --- | --- |
| Gene | Sequences | |
| *Myf5* -57.5 kb | F:ATACAGACATGCAGGCTTCAC | R:CTCCGTATGTTTGTTGGAAAGG |
| *Myf5* -0.7 kb | F:ACACGGCTCTTAAAGCAATGG | R: AACTGCTCTGACGGCATGGTA |
| *Myf5* -1.5 kb | F: ACAGCAGCTTTGACAGCATC | R: GGACAGACTGCCATGACTGA |
| *IgH* enhancer | F:CTGGACAGAGTGTTTCAAAAC | R: TGGCAGGAAGCAGGTCATGT |
